# Cardite de Lyme: Uma Causa Infecciosa de Bloqueio Atrioventricular – Relato de Caso

**DOI:** 10.36660/abc.20240301

**Published:** 2024-10-24

**Authors:** Eduardo Dan Itaya, Danilo Hantschick Fernandes Monteiro, Gabriela Coelho Itaya, Nathan Kong, Andre d’Avila

**Affiliations:** 1 University of Connecticut School of Medicine Division of Medicine Farmington Connecticut EUA University of Connecticut School of Medicine – Division of Medicine, Farmington, Connecticut – EUA; 2 Hospital SOS Cardio Florianópolis SC Brasil Hospital SOS Cardio, Florianópolis, SC – Brasil; 3 Harvard T H Chan School of Public Health Boston Massachusetts EUA Harvard T H Chan School of Public Health, Boston, Massachusetts – EUA; 4 Beth Israel Deaconess Medical Center Division of Cardiology Boston Massachusetts EUA Beth Israel Deaconess Medical Center – Division of Cardiology, Boston, Massachusetts – EUA; 5 Beth Israel Deaconess Medical Center Harvard-Thorndike Electrophysiology Institute Boston Massachusetts EUA Beth Israel Deaconess Medical Center – Harvard-Thorndike Electrophysiology Institute, Boston, Massachusetts – EUA

**Keywords:** Bloqueio Atrioventricular, Borrelia, Doença de Lyme

## Introdução

A doença de Lyme (DL) é a doença mais comum transmitida por carrapatos nos Estados Unidos, causada pela espiroqueta *Borrelia burgdorferi*. A apresentação mais comum é a erupção cutânea eritema migrans, observada em mais de 90% dos casos durante o estágio inicial localizado.^1,2^ Semanas ou meses após a picada do carrapato, a doença pode progredir para manifestações extracutâneas na fase disseminada inicial, levando a sintomas cardíacos, neurológicos e articulares.^2,3^

A Cardite de Lyme (CL) é uma complicação rara da DL, ocorrendo em 0,3% a 4% dos adultos não tratados.^4,5^ As manifestações mais proeminentes são distúrbios do sistema de condução envolvendo o nó atrioventricular (AV), frequentemente se apresentando como bloqueio AV (BAV) de alto grau.

Em 2021, os EUA relataram 24.610 casos confirmados e prováveis de DL.^6^ No entanto, a incidência é desconhecida – uma análise de banco de dados de reivindicações de seguros comerciais estimou 476.000 casos anuais nos EUA.^7^ Surpreendentemente, apesar das três regiões com maiores índices de DL estarem entre os destinos mais frequentes dos viajantes brasileiros,^8^ nenhum caso de CL foi relatado no Brasil. Isso destaca o potencial subdiagnóstico dessa condição em viajantes que retornam.

Portanto, conhecer essa etiologia infecciosa reversível do BAV é essencial para evitar tratamento tardio e intervenções desnecessárias, como implante de marcapasso permanente. Este relato de caso detalha um caso de CL caracterizado pela progressão eletrocardiográfica clássica e elucida o manejo dessa rara condição.

## Relato de Caso

Um homem de 43 anos apresentou um histórico de duas semanas de tontura intermitente, dor de cabeça posterior e uma fadiga fácil recente ao esforço. Inicialmente, ele desenvolveu uma dor de cabeça posterior e tontura, que depois melhoraram, mas depois progrediram para fadiga ao esforço. O paciente relatou exposição à natureza em Cape Cod, MA - Estados Unidos, nas últimas 2 semanas, mas não se lembra de picadas de carrapatos. Ao retornar, sua pressão arterial estava normal, mas revelou uma frequência cardíaca de cerca de 50 bpm, significativamente menor do que sua linha de base usual de 70 a 80 bpm. Essa descoberta levou a uma apresentação ao Pronto Socorro. Fora isso, ele negou dor no peito e falta de ar.

Apesar da infecção recente de um membro da família por COVID-19, o paciente testou negativo. O paciente supostamente tem um histórico remoto de DL, que foi tratada com antibióticos por várias semanas. Seu histórico médico anterior é significativo para pré-diabetes. Ele não tem histórico de doença cardiovascular, alergias ou uso diário de medicamentos. Além disso, não há histórico familiar de doença cardíaca ou morte súbita.

O exame físico revelou uma pressão arterial de 147/82 mm Hg e uma frequência cardíaca de 53 bpm. O paciente também tinha duas lesões planas na parte superior média direita das costas, consistentes com eritema crônico migratório. Um eletrocardiograma (ECG) inicial revelou um bloqueio cardíaco completo com dissociação AV. Um ECG repetido 30 minutos depois foi significativo para um BAV 2:1. A radiografia de tórax foi sem processo cardiopulmonar agudo, e os testes laboratoriais foram significativos apenas para transaminases elevadas (ALT: 251, AST: 92 e Fosfatase Alcalina: 185). Todos os outros testes, incluindo eletrólitos séricos e troponina, estavam dentro dos limites normais.

O paciente foi internado para avaliação adicional. Apesar da bradicardia contínua, ele manteve a perfusão sem nenhuma evidência de disfunção de órgão-alvo. Dada a ausência de comorbidades e exposição recente ao ar livre, ele começou a tomar Ceftriaxona intravenosa 2 g diariamente como tratamento empírico para o diagnóstico presuntivo de CL. Seu ecocardiograma transtorácico revelou tamanhos normais de cavidade biventricular, função sistólica biventricular regional e global normal e uma fração de ejeção do ventrículo esquerdo de 65%, sem patologia valvar ou fluxo patológico. A ultrassonografia do fígado revelou três hemangiomas sem evidência de hepatite ou esteatose. Uma ressonância magnética do fígado mostrou lesões consistentes com hemangiomas. A sorologia revelou anticorpos positivos para Lyme, Anasplasma negativo e um painel de hepatite negativo.

No segundo dia de internação, seu ECG foi significativo para BAV de 2º grau Mobitz tipo 1, com condução AV 2:1, frequência ventricular de 52 bpm e PR 196 ms. No dia seguinte, o ECG progrediu para BAV de 1º grau, com intervalo PR de 320 ms. Durante esse tempo, ele permaneceu hemodinamicamente e clinicamente estável.

Uma semana depois, ele recebeu alta com um plano para completar um curso de 21 dias de Ceftriaxona. O ECG de alta mostrou um ritmo sinusal com um atraso AV de 1º grau, com um intervalo PR de 222 ms e 63 bpm. Após um mês, durante uma visita de acompanhamento à clínica de eletrofisiologia, o paciente estava em ritmo sinusal, não teve eventos de monitoramento cardíaco e estava assintomático. Seu ECG mostrou um ritmo sinusal com um intervalo PR de 130 ms e 84 bpm.

## Discussão

**Figura 1:**
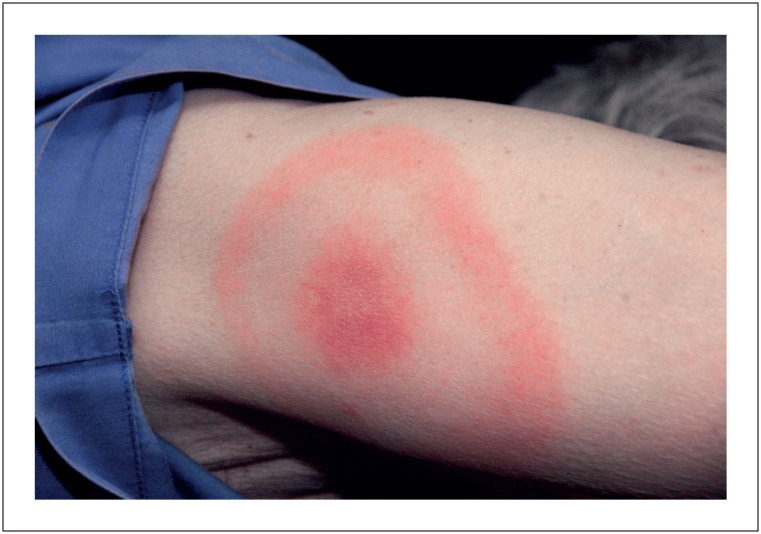
Eritema migrans, a erupção cutânea eritematosa patognomônica no padrão "erupção cutânea em alvo". Reproduzido de CDC / James Gathany, 2007.

A DL é causada pela espiroqueta *Borrelia burgdorferi*. A DL se tornou a principal doença transmitida por carrapatos na América do Norte e na Europa.^9^ Nossa complicação rara relatada, CL, ocorre em 0,3% a 4% dos adultos não tratados.^4,5^ Frequentemente se apresentando como AVB de alto grau. Esta complicação pode aparecer vários dias a meses após a picada do carrapato ou aparecimento de sintomas semelhantes aos da gripe e eritema migrans (Figura 1),^2,3,10^ observada em 90% dos casos de DL.^1,2^ Entretanto, estudos epidemiológicos sugerem que apenas 40% dos pacientes com CL se lembram dessas lesões cutâneas características.^4,11^

Acredita-se que o mecanismo desta doença surja da invasão direta do miocárdio pela bactéria^5^ e uma resposta imune exagerada subsequente.^3^ Acredita-se que a resposta imune seja secundária a uma reação cruzada entre antígenos de *B. burgdorferi* e epítopos cardíacos.^12^ Este processo inflamatório é composto principalmente por macrófagos e linfócitos,^13^ resultando em uma lesão autoimune.^12^

Assim como no nosso caso apresentado, o BAV é a apresentação mais comum de CL, ocorrendo em até 90% dos casos.^5^ Esse fenômeno é tipicamente caracterizado por mudanças nos graus de BAV ao longo de dias, horas ou até minutos.^3^ Corroborando nosso caso, em que o ECG inicial revelou bloqueio cardíaco completo com dissociação AV, e aproximadamente 30 minutos depois, progrediu para um BAV 2:1. A progressão para um BAV completo pode ocorrer rapidamente e ser fatal.^14^ Portanto, os pacientes com CL devem ser submetidos a monitoramento contínuo de telemetria e ECG.

A antibioticoterapia deve ser iniciada assim que houver suspeita clínica de CL para diminuir a duração da doença e prevenir complicações.^15^ O prognóstico é favorável, com resolução do BAV de alto grau geralmente após 10 dias de tratamento com antibióticos.^5^ Em pacientes com CL grave – BAV de 1º grau sintomático com intervalo PR ≥ 300 mseg, BAV de 2º ou 3º grau –^16^ Ceftriaxona IV empírica 2g, uma vez ao dia, deve ser administrada imediatamente. Após a confirmação, o tratamento com antibióticos deve continuar de 14 a 21 dias com base na gravidade da apresentação.^3^ A Tabela 1 resume o tratamento da CL em adultos.^16^

**Tabela 1:** Tratamento com antibióticos para CL em adultos

	Antibiótico	Dose	Duração
**Forte**			
	Ceftriaxona	2 g IV uma vez ao dia	14 – 21 dias*
**Leve**			
	Doxiciclina	100 mg oral, duas vezes ao dia	14 – 21 dias
	Amoxicilina	500 mg por via oral, três vezes ao dia	
	Cefuroxima	500 mg por via oral, duas vezes ao dia	

Grave: sintomático, BAV de 1º grau com PR ≥ 300 ms, BAV de 2º ou 3º grau. Leve: BAV de 1º grau com PR < 300 ms.

* Após a resolução dos sintomas e do BAV de alto grau, considere a transição para antibióticos orais para completar o tratamento. BAV: bloqueio atrioventricular; IV: intravenoso; ms: milissegundos.

**Figura 2:**
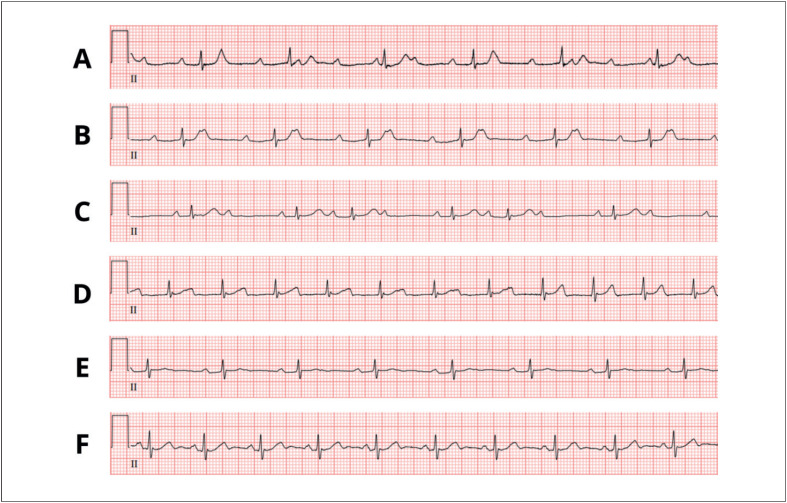
Progressão eletrocardiográfica da Cardite de Lyme. (A) BAV de terceiro grau com ritmo de escape juncional (dia 1). (B) BAV 2:1 (dia 1 - 30 minutos depois). (C) BAV de segundo grau (Mobitz I) com condução AV 2:1 (dia 3). (D) Atraso de BAV de primeiro grau com intervalo PR de 320 ms (dia 4). (E) BAV de primeiro grau com intervalo PR de 222 ms (dia 8). (F) Ritmo sinusal (acompanhamento de 1 mês). BAV: bloqueio atrioventricular; ms: milissegundos.

Um achado interessante da CL é que o distúrbio de condução geralmente se resolve de forma gradual, do BAV de 3º grau ao bloqueio de Wenckebach de 2º grau, ao bloqueio de 1º grau, à diminuição do intervalo PR e ao ECG normal.^1,15^ Esse achado também estava presente em nosso paciente, conforme representado na Figura 2.

Embora a estimulação permanente continue sendo o tratamento padrão para BAV de alto grau, a natureza variável da CL e a resolução completa das alterações de ECG com antibioticoterapia na maioria dos pacientes apoiam o uso de estimulação temporária se houver evidência de disfunção de órgão terminal ou instabilidade clínica.^5,13,17^ Dado que um terço dos pacientes necessita de estimulação temporária,^15,17,18^ essa abordagem reduz intervenções desnecessárias e ajuda a mitigar riscos potenciais associados à implantação de marcapasso. Em uma revisão sistemática de casos de CL, Besant et al. revelaram que um marcapasso permanente foi colocado em 12,5% dos pacientes, e mais da metade desses pacientes tiveram uma reversão do BAV com tratamento com antibióticos.^18^

Além disso, a CL afeta desproporcionalmente um grupo demográfico jovem, normalmente com idades entre 20 e 40 anos,^19^ o que aumentaria ainda mais a exposição a complicações ao longo da vida, como a necessidade de múltiplas trocas de gerador, e os riscos associados, incluindo infecções, deslocamento de cabos, custos de assistência médica e até mesmo sequelas psicológicas e físicas.^5^ Entretanto, um marcapasso permanente deve ser considerado se a condução AV 1:1 não for restaurada dentro de 14 dias após a admissão.^3^

O diagnóstico é determinado por meio de um ensaio imunoenzimático para triagem de anticorpos IgM e IgG, seguido de confirmação usando um ensaio Western Blot para resultados positivos ou limítrofes. IgM aparece dentro de uma a duas semanas, seguido por IgG dentro de duas a seis semanas após a manifestação cutânea.^20^ A alta variabilidade do antígeno Borrelia afeta a sensibilidade e especificidade da sorologia. No entanto, a sensibilidade é alta quando manifestações extracutâneas estão presentes, como durante a fase disseminada inicial quando ocorre CL.^3^

Portanto, a CL é essencial como diagnóstico diferencial de BVA, especialmente em pacientes jovens com histórico de atividade ao ar livre em áreas endêmicas, incluindo as regiões Nordeste, Centro-Atlântico e Centro-Oeste dos EUA.^19^

## Conclusão

Este relato de caso ilustra um caso clássico de CL, destacando a importância crítica de reconhecer essa etiologia infecciosa de distúrbios do sistema de condução, afetando notavelmente o nó AV. Embora não tenha havido casos relatados no Brasil, a DL continua sendo um diagnóstico diferencial crucial para BAV de alto grau em viajantes que retornam de áreas endêmicas. Portanto, como demonstrado em nosso caso, a conscientização e o conhecimento da CL são essenciais para que os clínicos garantam cuidados oportunos e apropriados e evitem intervenções desnecessárias.
