# Supplementary material for: Cardite de Lyme: Uma Causa Infecciosa de Bloqueio Atrioventricular – Relato de Caso
Source: Arq Bras Cardiol. 2024 Oct 24;121(10):e20240301. [Article in Portuguese] doi: 10.36660/abc.20240301 (PMC11634212; doi:10.36660/abc.20240301)
Supplement: Translation

# Translation: Lyme Carditis: An Infectious Cause of Atrioventricular Block – A Case Report

**DOI:** 10.36660/abc.20240301i

**Authors:** Eduardo Dan Itaya, Danilo Hantschick Fernandes Monteiro, Gabriela Coelho Itaya, Nathan Kong, Andre d’Avila

**Affiliations:** 1 Farmington Connecticut USA University of Connecticut School of Medicine – Division of Medicine, Farmington, Connecticut – USA; 2 Florianópolis SC Brazil Hospital SOS Cardio, Florianópolis, SC – Brazil; 3 Boston Massachusetts USA Harvard T H Chan School of Public Health, Boston, Massachusetts – USA; 4 Boston Massachusetts USA Beth Israel Deaconess Medical Center – Division of Cardiology, Boston, Massachusetts – USA; 10 Boston Massachusetts USA Beth Israel Deaconess Medical Center – Harvard-Thorndike Electrophysiology Institute, Boston, Massachusetts – USA

**Keywords:** Atrioventricular Block, Borrelia, Lyme Disease

## Introduction

Lyme disease (LD) is the most common tick-borne illness in the United States, caused by the spirochete *Borrelia burgdorferi*. The most common presentation is the erythema migrans skin rash, observed in over 90% of cases during the early localized stage.^1,2^ Weeks to months post-tick bite, the disease may progress to extracutaneous manifestations in the early disseminated phase, leading to cardiac, neurologic, and joint symptoms.^2,3^

Lyme carditis (LC) is a rare complication of LD, occurring in 0.3% to 4% of untreated adults.^4,5^ The most prominent manifestations are conduction system disturbances involving the atrioventricular (AV) node, often presenting as high-degree AV block (AVB).

In 2021, the US reported 24,610 confirmed and probable cases of LD.^6^ However, the incidence is unknown – a commercial insurance claims database analysis estimated 476,000 annual cases in the US.^7^ Surprisingly, despite the three regions with the highest rates of LD being among the most frequent destinations of Brazilian travelers,^8^ no cases of LC have been reported in Brazil. This highlights the potential underdiagnosis of this condition in returning travelers.

Therefore, knowing this reversible infectious etiology of AVB is essential to prevent delayed treatment and unnecessary interventions, such as permanent pacemaker implantation. This case report details an LC case characterized by the classic electrocardiographic progression and elucidates the management of this rare condition.

## Case Report

A 43-year-old male presented with a two-week history of intermittent lightheadedness, posterior headache, and a recent easy fatiguability on exertion. Initially, he developed a posterior headache and lightheadedness, which then improved but later progressed to experiencing fatigue on exertion. The patient reported exposure to nature in Cape Cod, MA – United States, in the previous 2 weeks but has no recollection of tick bites. Upon his return, his blood pressure was normal but revealed a heart rate of around 50 bpm, significantly lower than his usual baseline of 70 to 80 bpm. This finding prompted a presentation to ED. Otherwise, he denied chest pain and shortness of breath.

Despite a family member's recent COVID-19 infection, the patient tested negative. The patient reportedly has a remote history of LD, which was treated with antibiotics over several weeks. His past medical history is significant for prediabetes. He has no cardiovascular disease history, allergies, or daily medication use. Additionally, there is no family history of cardiac disease or sudden death.

Physical examination revealed a blood pressure of 147/82 mm Hg and a heart rate of 53 bpm. The patient also had two flat lesions on the mid-right upper back, consistent with erythema chronicum migrans. An initial electrocardiogram (ECG) revealed a complete heart block with AV dissociation. A repeat ECG 30 minutes later was significant for a 2:1 AVB. The chest radiography was without acute cardiopulmonary process, and laboratory tests were only significant for elevated transaminases (ALT: 251, AST: 92, and Alkaline Phosphatase: 185). All other tests, including serum electrolytes and troponin, were within normal limits.

The patient was admitted for further evaluation. Despite ongoing bradycardia, he maintained perfusion without any evidence of end-organ dysfunction. Given the absence of comorbidities and recent outdoor exposure, he was started on intravenous Ceftriaxone 2 g daily as empiric treatment for the presumptive diagnosis of LC. His transthoracic echocardiogram revealed normal biventricular cavity sizes, normal regional and global biventricular systolic function, and a left ventricular ejection fraction of 65%, without valvular pathology or pathologic flow. Liver Ultrasound revealed three hemangiomas without evidence of hepatitis or steatosis. A liver MRI showed lesions consistent with hemangiomas. Serology revealed positive Lyme antibodies, negative Anasplasma, and a negative hepatitis panel.

On his second day of admission, his ECG was significant for 2nd-degree AVB Mobitz type 1, with 2:1 AV conduction, ventricular rate of 52 bpm, and PR 196 ms. The following day, the ECG progressed to a 1st-degree AVB, with a PR interval of 320 ms. During this time, he remained hemodynamically and clinically stable.

One week later, he was discharged with a plan to complete a 21-day course of Ceftriaxone. The discharge ECG showed a sinus rhythm with a 1st-degree AV delay, with a PR interval of 222 ms and 63 bpm. After one month, during a follow-up visit to the electrophysiology clinic, the patient was in sinus rhythm, had no cardiac monitoring events, and was asymptomatic. His ECG showed a sinus rhythm with a PR interval of 130 ms and 84 bpm.

## Discussion

LD is caused by the spirochete *Borrelia burgdorferi*. LD has become the leading tick-borne illness in North America and Europe.^9^ Our reported rare complication, LC, occurs in 0.3% to 4% of untreated adults.^4,5^ Often presenting as high-degree AVB. This complication can appear several days to months after the tick bite or appearance of flu-like symptoms and erythema migrans (Figure 1),^2,3,10^ observed in 90% of LD cases.^1,2^ However, epidemiologic studies suggest that only 40% of patients with LC recall these characteristic skin lesions.^4,11^

**Figure 1:** Erythema migrans, the pathognomonic erythematous rash in the "bulls-eye rash" pattern. Reproduced from CDC / James Gathany, 2007.

The mechanism of this disease is thought to arise from direct myocardial invasion by the bacteria^5^ and a subsequent exaggerated immune response.^3^ The immune response is hypothesized to be secondary to a cross-reaction between *B. burgdorferi* antigens and cardiac epitopes.^12^ This inflammatory process is mainly composed of macrophages and lymphocytes,^13^ resulting in an autoimmune injury.^12^

As in our presented case, AVB is the most common presentation of LC, occurring in up to 90% of cases.^5^ This phenomenon is typically characterized by changing degrees of AVB over days, hours, or even minutes.^3^ Corroborating our case, in which the initial ECG revealed a complete heart block with AV dissociation, and approximately 30 minutes later, it progressed to a 2:1 AVB. The progression to a complete AVB can occur rapidly and be fatal.^14^ Thus, LC patients should undergo continuous telemetry and ECG monitoring.

Antibiotic therapy must be started as soon as there is a clinical suspicion of LC to decrease the disease's duration and prevent complications.^15^ The prognosis is favorable, with resolution of the high-degree AVB generally after 10 days of antibiotic treatment.^5^ In patients with severe LC – symptomatic, 1st-degree AVB with PR interval ≥ 300 msec, 2nd or 3rd-degree AVB –^16^ empiric IV Ceftriaxone 2g, once a day, should be administered immediately. After confirmation, antibiotic treatment should continue from 14 to 21 days based on the severity of the presentation.^3^ Table 1 summarizes the treatment of LC in adults.^16^

**Table 1:** Antibiotic Treatment for LC in Adults

	Antibiotic	Dose	Duration
**Severe**			
	Ceftriaxone	2 g IV once daily	14 – 21 days*
**Mild**			
	Doxycycline	100 mg oral, twice per day	14 – 21 days
	Amoxicillin	500 mg oral, three times per day	
	Cefuroxime	500 mg oral, twice per day	

Severe: symptomatic, 1st degree AVB with PR ≥ 300 ms, 2nd or 3rd degree AVB. Mild: 1st degree AVB with PR < 300 ms.

* After the resolution of symptoms and high-degree AVB, consider transitioning to oral antibiotics to complete the treatment course. AVB: atrioventricular block; IV: intravenous; ms: milliseconds.

An interesting finding of LC is that conduction disturbance usually resolves in a stepwise manner, from 3rd-degree AVB to Wenckebach 2nd-degree block, to 1st-degree block, to decreasing PR interval, to a normal ECG.^1,15^ This finding was also present in our patient, as represented in the Figure 2.

**Figure 2:** Electrocardiographic progression of Lyme Carditis. (A) Third-degree AVB with a junctional escape rhythm (day 1). (B) 2:1 AVB (day 1 - 30 minutes later). (C) Second-degree AVB (Mobitz I) with 2:1 AV conduction (day 3). (D) First-degree AVB delay with PR interval of 320 ms (day 4). (E) First-degree AVB with PR interval of 222 ms (day 8). (F) Sinus rhythm (follow-up 1 month). AVB: atrioventricular block; ms: milliseconds.

While permanent pacing remains the standard treatment for high-degree AVB, the variable nature of LC and the complete resolution of ECG alterations with antibiotic therapy in most patients support the use of temporary pacing if there is evidence of end-organ dysfunction or clinical instability.^5,13,17^ Given that one-third of patients require temporary pacing,^15,17,18^ this approach reduces unnecessary interventions and helps mitigate potential risks associated with pacemaker implantation. In a systematic review of LC cases, Besant et al. revealed that a permanent pacemaker was placed in 12.5% of patients, and more than half of those patients had a reversal of AVB with antibiotic treatment.^18^

Additionally, LC disproportionately affects a young demographic, normally aged 20–40 years,^19^ which would further increase the exposure to lifetime complications, such as the need for multiple generator changes, and the associated risks, including infections, lead dislodgement, healthcare costs, and even psychological and physical sequelae.^5^ However, a permanent pacemaker should be considered if 1:1 AV conduction is not restored within 14 days post-admission.^3^

The diagnosis is determined through an enzyme-linked immunosorbent assay to screen for IgM and IgG antibodies, followed by confirmation using a Western blot assay for positive or borderline results. IgM appears within one to two weeks, followed by IgG within two to six weeks after the cutaneous manifestation.^20^ The Borrelia antigen's high variability affects the serology's sensitivity and specificity. However, the sensitivity is high when extracutaneous manifestations are present, such as during the early disseminated phase when LC occurs.^3^

Therefore, LC is essential as a differential diagnosis of AVB, especially in young patients with a history of outdoor activity in endemic areas, including the US Northeast, mid-Atlantic, and upper Midwest regions.^19^

## Conclusion

This case report illustrates a classic case of LC, highlighting the critical importance of recognizing this infectious etiology of conduction system disturbances, notably affecting the AV node. Although there have been no reported cases in Brazil, LD remains a crucial differential diagnosis for high-degree AVB in travelers returning from endemic areas. Therefore, as demonstrated in our case, awareness and knowledge of LC are essential for clinicians to ensure timely and appropriate care and prevent unnecessary interventions.
